# Case Report of an Endovascular Repair of a Residual Type A Dissection Using a Not CE Not FDA-Approved Najuta Thoracic Stent Graft System

**DOI:** 10.1097/MD.0000000000000436

**Published:** 2015-01-26

**Authors:** N. Mangialardi, S. Ronchey, A. Malaj, M. Lachat, E. Serrao, V. Alberti, S. Fazzini

**Affiliations:** From the Department of Vascular Surgery (NM, SR, ES, VA, SF), San Filippo Neri Hospital; Department of Vascular Surgery (AM), Policlinico Umberto I, Rome, Italy; and Clinic for Cardiovascular Surgery (ML), University Hospital Zurich, Zurich, Switzerland.

## Abstract

This report describes an endovascular repair of a residual type A dissection using a medical device that is not marked by european conformity (CE) or Food and Drug Administration (FDA).

The patient underwent ascending aortic surgery for acute type A dissection. The 2-year angio–computed tomography demonstrated patency of the residual false lumen with evolution into a 6 cm aneurysm, the extension of the dissection from the aortic arch to the aortic bifurcation with thrombosis of the right common iliac artery. There was no CE- or FDA-marked medical device indicated for this case or any other acceptable therapeutic alternative.

We used the Najuta thoracic stent graft and successfully handled the pathology in a multiple-phase treatment.

Technology is evolving with specific grafts for the ascending and fenestrated grafts for the aortic arch. In this single case the Najuta endograft, in spite of the periprocedural problems, was a valid therapeutic option.

## INTRODUCTION

The close follow-up of operated-on acute type A aortic dissection survivors is important.^1^ The long-term mortality rate is higher than that of the normal population and the outcomes may serve as a benchmark for catheter-based (percutaneous, endovascular) and hybrid treatment modalities.

We report the treatment of a residual type A dissection using a “custom-made” fenestrated endograft. In this case all companies that commercialize custom-made endografts in EU refused, for different reasons, the production of an adequate endoprosthesys. The comorbidities of the patient did not allow us to attempt any other way of treatment and so we decided to ask Kawasumi Laboratories in Tokyo, Japan, for a custom-made device.

Kawasumi Laboratories develop and produce the Najuta endograft system.^2^ These grafts are approved by the authority under applicable laws in Japan, but have no European conformance (CE) or Food and Drug Administration (FDA) approval. After receiving the necessary permissions, we handled the pathology in a multiple-phase treatment, using the “Najuta” stent graft. We think by reporting this case, we are adding another element in the large field, and not just a chapter, of the aortic arch pathologies and their treatment.

## MATERIALS AND METHODS

A 57-year-old Jehovah's Witness gentleman was hospitalized in 2011 in another facility for acute type A dissection and underwent replacement of the ascending aorta from the sinotubular ridge to the origin of the innominate artery with a Dacron tube graft followed by pacemaker implantation. A computed tomography (CT) angiography exam demonstrated the patency of the false lumen, the extension of the dissection to the aortic arch, descending aorta, and aortic bifurcation with thrombosis of the right common iliac artery. At that time the cardiac surgeon decided for a medical treatment of the residual dissection.

**Figure 1:**
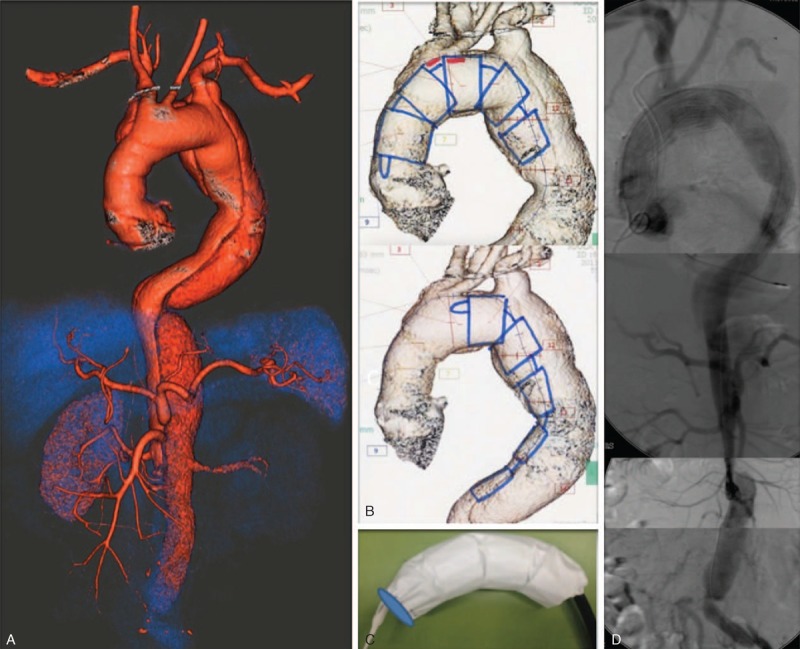
(A) Volume rendering of the residual type A dissection; (B) Najuta endograft; (C) precurved shape of the Najuta endograft; (D) reconstruction of the final angiography control.

On July 2013 the patient came under our observation with a CT control demonstrating the evolution into a 6 cm arch aneurysm of the false lumen. The celiac trunk and the superior mesenteric and right renal arteries were perfused by the true lumen; the left renal artery was perfused by the false lumen (Figure 1A).

The patient's comorbidities (severe chronic obstructive pulmonary disease, obstructive sleep apnea syndrome, obesity, prior sternotomy, and hypertension) made even more high risk an already technically complex surgical reoperation to replace the aortic arch and the descending thoracic aorta. He was classified as an ASA IV patient. We decided to treat his pathology in an endovascular fashion with a “custom-made” fenestrated endograft.

We contacted the companies that owned CE- or FDA-marked devices for a fenestrated custom-made device but they refused for different reasons: limited clinical access for their devices to a small group of physicians as they were gaining experience with their grafts and developing an understanding of the patients who may benefit from their use or because it was impossible to manufacture a specific graft for this case. At present there is no other CE-marked endovascular device compatible with the indications of the case or any other FDA-marked device.

We contacted Kawasumi Laboratories in Tokyo, Japan, and asked them to consider the treatment of this patient with the Najuta endograft system (Figure 1B).

The Najuta endograft system is designed for thoracic aneurysm repair and has been developed at the Tokyo Medical University. It is a semi–custom-made stent graft having 950 patterns of 3-dimensional structures for thoracic aortic aneurysm, which enables to provide a suitable stent graft matching with a patient's anatomy of the aorta by applying detailed image analysis. The basic architecture of the Najuta system includes a rigid graft column structure that apposes the curvature of the aorta to prevent endograft migration as well as postoperative endoleaks. It has a precurved shape, which is critical for enhancing the stability of the endograft in the aortic arch (Figure 1C). Z-stent elements are connected with longitudinal support struts and covered with polytetrafluoroethylene (PTFE). The strategic location of these struts is designed to allow the stent position alignment to adjust with the straight portion of the aorta. Because the PTFE graft material is sutured to the stent framework at both ends of the endograft only, even if the stent is deformed or kinked due to tortuous anatomy, the PTFE will be able to expand to its diameter, thereby maintaining the luminal area. It requires fewer suturing between Z-stent and graft, because the connecting struts support the shape of precurved skeletons. This device does not have a high radial force.^3,4^ “Najuta” stent grafts are approved by the authority under applicable laws in Japan and are not approved for aortic dissection, but in this case they were used to treat a residual type A dissection. Informed consent from the patient was acquired in which the description of the medical device, the lack of the CE marking, the reasons for which the patient was not a candidate for the traditional therapeutic treatment, information on potential adverse events, and the absence of similar therapeutic alternatives were clearly spelled out.

The stent graft studied for the patient deviated from a “Najuta” stent grafts; it had a single fenestration at the center, which was one of the differences when compared with the normal design.

## Procedure

The procedure was performed under general anesthesia. The left femoral artery was exposed and used as the access vessel and percutaneous access was performed to the right brachial artery. We used a 24F Gore DrySeal (W.L. Gore & Associates, Flagstaff, AZ) introducer sheath at the left common femoral artery and a 7F Cook Flexor 55 cm (Cook Incorporated, Bloomington, IN) introducer sheath at the right brachial artery.

From the brachial artery, with a Bern catheter (Boston Scientific Corporation, Natick, MA) and a 0.035 Terumo standard wire (Tokyo, Japan), we fenestrated the intimal flap at the abdominal level to ensure an adequate left renal artery perfusion after the coverage of the proximal entry tear, and then we pulled downward to the true lumen the Terumo standard wire (Tokyo, Japan) promptly recuperated with an Amplatz GooseNeck (CONVIDIEN, Plymouth, MN, US) at the left iliac artery. We had to change the 0.035 through-and-through wire for a 0.032, 400 cm Terumo standard wire because the Najuta endograft was not fit for a 0.035 guidewire. In this way, using this teleferic guide from the right brachial artery to the left femoral artery, we introduced and released in a retrograde way the 2 modules of the endoprosthesys, a distal tapered module 34 to 30 × 200 mm and a proximal one 34 × 200 mm. The correct position of the device in the arch was easily obtained, thanks to a precurved shape specifically designed for the aortic arch of the patient; the fenestration was automatically rotated and oriented toward the supra-aortic branches. The proximal landing zone was about 2 cm distal to the sinotubular ridge. There is no need for cardiac pacing during the deployment, because the graft opens with a wind sock effect taking advantage of the cardiac output. The final angiography confirmed the proper positioning of the device and the absence of endoleaks, the exclusion of the proximal entry tear, and the patency of the brachiocephalic artery and the left carotid artery (Figure 1D); also the celiac trunk and the superior mesenteric and right renal arteries were well perfused by the true lumen; the left renal artery was perfused by the false lumen via the fenestration at the abdominal level.

**Figure 2:**
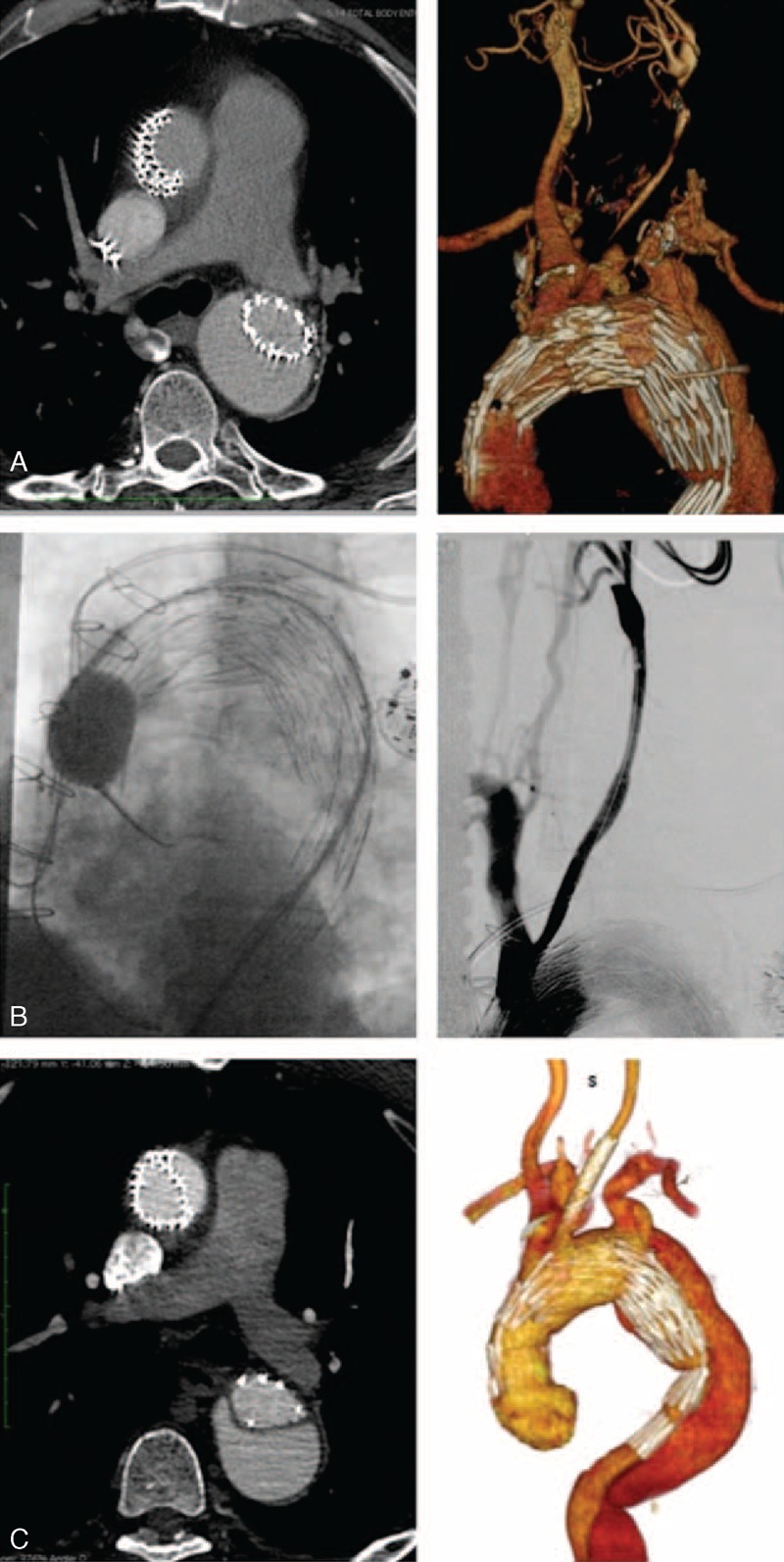
(A) Angio-CT documenting the proximal infolding of the endograft and the left common carotid dissection; (B) angioplasty of the proximal infolding and retrograde angiography of the dissected left common carotid; (C) angio-CT documenting the residual proximal leak and no residual dissection of the left common carotid. CT = computed tomography.

One week after the procedure, the patient presented recurrent transient ischemic attacks (TIAs) with right arm impairment and speech disturbances. A left common carotid dissection was detected by color-flow mapping and confirmed by CT sequences. The angio-CT showed also an asymptomatic proximal infolding of the endograft causing a type 1A endoleak (Figure 2A). So we proceeded with the second surgery step. The left femoral artery was easily reexposed and the true lumen was regained. At that time we did not have disponibility of an adequate aortic stent; therefore, the proximal infolding of the endograft was treated by simple angioplasty inflating a Coda Balloon 40 mm (Cook Incorporated) (Figure 2B). The left common carotid artery was exposed and, in a retrograde fashion, a Carotid WALLSTENT (Boston Scientific Corporation) was released at the origin of the left common carotid artery. Angiography demonstrated the optimal position of the stent and no residual dissection of the left carotid. The procedure was successful and the patient did not experience further TIA, but the angio-CT control revealed a residual proximal leak (Figure 2C).

**Figure 3:**
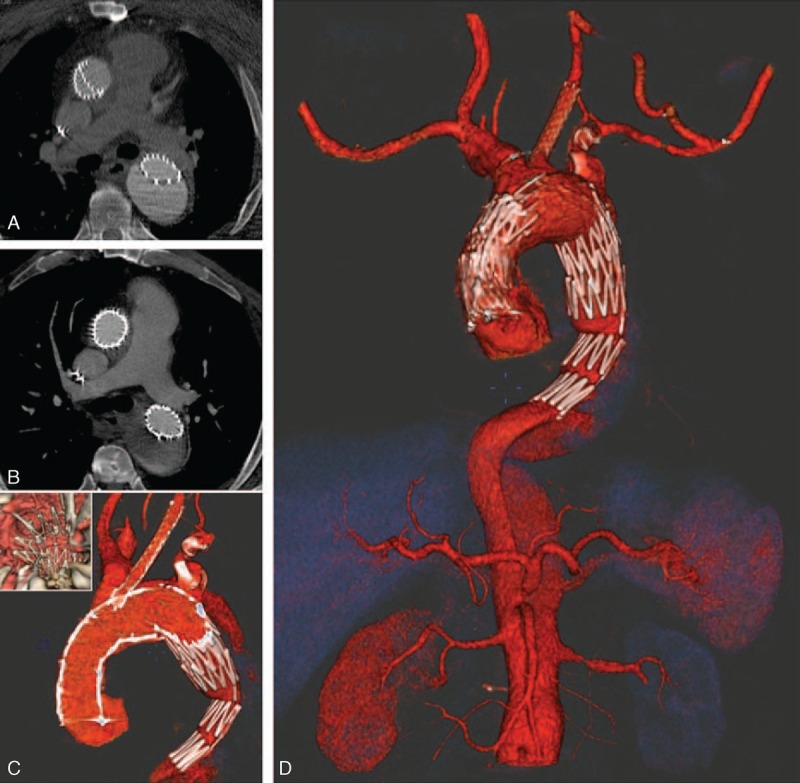
(A) Angio-CT documenting the evolution of the proximal infolding; (B) angio-CT documenting the resolution of the proximal infolding by the well-expanded Jotec stent; (C) final volume rendering of the arch; (D) final volume rendering of the aorta. CT = computed tomography.

One month later the patient returned for the third surgery step, referring also left arm claudication. Percutaneous access was performed to the left femoral artery and the true lumen was regained. We introduced and released an aortic stent, Jotec “double flared” 32 to 70 mm (Hechingen, Germany), at the proximal landing zone (Figure 3A) of the Najuta endograft. Angiography demonstrated the optimal position of the stent, no residual infolding, and the patency of both coronary ostia. The left femoral artery puncture site was sutured using 2 Perclose ProGlide systems (Abbott Vascular Inc, Redwood City, CA).

Then we performed the left carotid-axillary artery bypass using a 7 mm ringed eparinated polytetrafluoroethylene prosthesis tunneled under the clavicle and the occlusion of the origin of the left subclavian artery with an Amplatzer vascular plug (St. Jude Medical, Plymouth, MN).

We decided to perform a carotid-axillary bypass^5^ and not the traditional carotid subclavian bypass due to the habitus of the patient and to avoid complications with the lymphatic channels and nerves more commonly exposed to lesions after a supraclavicular exposure. Final angio-CT, performed 1 month later, documented the good position of all the endografts and no residual infolding (Figure 3B–D).

## DISCUSSION

Thoracic endovascular aortic repair (TEVAR) has been established as the preferential therapeutic modality for the treatment of intact aneurysms of the descending thoracic aorta.^6^ Its application has also gained a wide acceptance for the treatment of acute thoracic aortic pathologies.^7^

But endografting in the aortic arch is associated with a higher morbidity and mortality than in the descending aorta pathologies. Recently endovascular debranching of the aortic arch with chimney stents has provided a minimally invasive alternative to open surgery with readily available devices.^8^

Most of the zone 0 intervention group still requires a thoracotomy and also the current choice of treatment of the ascending aorta is open graft replacement with or without aortic valve replacement, but approximately 20% of patients are considered unfit for open surgery.^9^ In the past few years, there has been a growing interest in the use of endografts in the ascending aorta.^10^ However, the biomechanical challenges of the ascending aorta remain formidable and the durability of endovascular repair in this territory is unknown. The ascending aorta is constantly subjected to large fluid and tissue stresses that are transmitted from the left ventricle. The short aortic length and the size discrepancy between proximal and distal segments create further challenges for designing devices that conform to its curvature while withstanding migratory forces. The biomechanical displacement of the ascending aorta is significantly greater than the arch and descending thoracic aorta, with an associated high rotational component. There is considerable risk of endograft migration, infolding, endoleak, or graft material failure. Iliopoulos et al^11^ found that wall stresses were greater in the circumferential rather than longitudinal direction in ascending aortic aneurysms. This may also explain why ascending aortic dissection is a common result of a circumferential tear in the intimal layer.

Brain injury after TEVAR is a major complication and most often associated with the underlying pathology or excessive device manipulation within the arch.^12,13^ Spinal cord injury can occur immediately after TEVAR or be delayed, requiring clinical and neurological surveillance.^14^ Retrograde aortic dissection may also occur, but in this case it was impossible due to the presence of the Dacron tube graft replacing the ascending aorta.

Stent graft–induced new entry has been increasingly observed after TEVAR of aortic dissection^15^ and the use of tapered stent grafts is of a great help to prevent them.

In this case we had no possibility to implant a CE- or FDA-approved stent graft and the general conditions of the patient and the refusal of any kind of blood transfusion did not permit a new open procedure to replace the aortic arch and the descending aorta. So we considered the treatment with the Najuta thoracic stent graft.

In this single case the Najuta endograft, in spite of the periprocedural problems, was a valid therapeutic option.
